# On the realistic contribution of European forests to reach climate objectives

**DOI:** 10.1186/s13021-019-0123-y

**Published:** 2019-06-14

**Authors:** Giacomo Grassi, Alessandro Cescatti, Robert Matthews, Gregory Duveiller, Andrea Camia, Sandro Federici, Jo House, Nathalie de Noblet-Ducoudré, Roberto Pilli, Matteo Vizzarri

**Affiliations:** 1 European Commission, Joint Research Centre, 21027 Ispra, VA Italy; 2 Forest Research, Alice Holt Lodge, Farnham, GU10 4LH UK; 3 Climate and Environment Division, FAO, 00153 Rome, Italy; 4 Department of Geographical Sciences, Cabot Institute, University of Bristol, Bristol, BS8 1SS UK; 5 Laboratoire des Sciences du Climat et de l’Environnement LSCE/IPSL, Unité mixte CEA-CNRS-UVSQ, Université Paris-Saclay, 91191 Gif-sur-Yvette, France

**Keywords:** EU climate target, Forest mitigation, GHG emissions, Biophysical effects, Bioenergy

## Abstract

A recent article by Luyssaert et al. (Nature 562:259–262, 2018) analyses the climate impact of forest management in the European Union, considering both biogeochemical (i.e., greenhouse gases, GHG) and biophysical (e.g., albedo, transpiration, etc.) effects. Based on their findings, i.e. that additional net overall climate benefits from forest management would be modest, the authors conclude that the EU “should not rely on forest management to mitigate climate change”. We first explain that most of the additional EU GHG mitigation effort by 2030 is expected to come from emission reductions and only a very small part from forestry, even when forest bioenergy is allowed for. Nevertheless, the inclusion of forest management in climate change mitigation strategies is key to identifying the country-specific optimal mix, in terms of overall GHG balance, between strategies focused on conserving and/or enhancing the sink and strategies focused on using more wood to reduce emissions in other GHG sectors. Then, while acknowledging the importance that biophysical effects have on the climate, especially at the local and seasonal scale, we argue that the net annual biophysical climate impact of forest management in Europe remains more uncertain than the net CO_2_ impact. This has not been adequately emphasized by Luyssaert et al. (2018), leading to conclusions on the net overall climate impact of forest management that we consider premature and applied to a partially biased perception of European policy towards forestry and climate change. To avoid further confusion in the debate on how forestry may contribute to mitigating climate change, a more constructive dialogue between the scientific community and policy makers is needed.

**Electronic supplementary material:**

The online version of this article (10.1186/s13021-019-0123-y) contains supplementary material, which is available to authorized users.

## Background

A recent article by Luyssaert et al. [1] analyses the climate impact of forest management in the European Union (EU) considering both biogeochemical (i.e., greenhouse gases, GHG) and biophysical (e.g., albedo, transpiration, etc.) effects. The context of the paper is the EU’s climate target under the Paris Agreement, i.e. a 40% reduction in GHG emissions by 2030 compared to 1990 levels (equivalent to a reduction of about 2250 Mt CO_2_e/year). In the original version of the paper, a key premise was that *“about 75% of this reduction is expected to come from emission reductions and the remaining 25% from land use, land*-*use change and forestry*”, citing Grassi et al. [2]. Based on their findings, i.e. that additional net climate benefits from forest management would be modest, Luyssaert et al. [1] conclude that the EU “*should not rely on forest management to mitigate climate change*”.

**Fig. 1:**
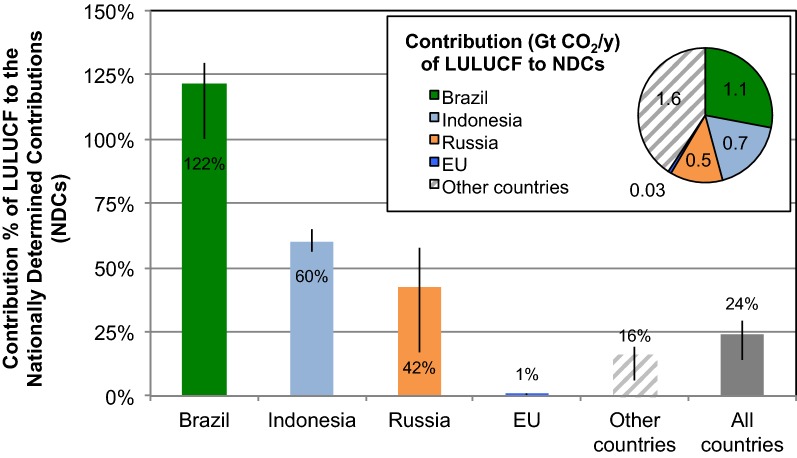
Contribution of LULUCF to the Nationally Determined Contributions (NDCs) in 2030 of Brazil, Indonesia, Russia and the EU, based on the analysis of Grassi et al. [2] expressed in % of the total GHG emissions reduction (main graph) and in GtCO_2_/year (small graph). The original estimate by Grassi et al. [2] for the EU (zero) is updated here to consider the recent EU LULUCF legislation [7] that caps the contribution from LULUCF toward the EU target at 280 MtCO_2_e for the 10-years period 2021–2030: if this value is annualized (i.e., 28 MtCO_2_e/year), it corresponds to slightly more than 1% of the EU 2030 emission reduction target (which is about 2250 Mt CO_2_e/year, i.e. from about 5650 Mt CO_2_e/year in 1990 to about 3400 Mt CO_2_e/year in 2030). More information on the NDCs is in Additional file 1: Table S1. For further details, see Fig. 4b, supplementary section 2 and supplementary Table 5 in Grassi et al. [2]

The original premise of Luyssaert et al. [1] on the expected large role of forestry in meeting the EU climate targets reflected a misinterpretation of Grassi et al. [2]. In fact, Grassi et al. [2] assume that the portion of the EU GHG mitigation target contributed by the land use, land-use change and forestry (LULUCF) sector is zero, consistent with [3]. The value of 25% refers to the globally aggregated contribution from LULUCF to the Nationally Determined Contributions made in Paris, mostly associated with the reduction of deforestation expected in the 2030 climate targets of Brazil and Indonesia (see Fig. 1 and Additional file 1: Section S1). This mistake has been acknowledged by Luyssaert et al. and a subsequent correction was published [4].

In this commentary, we discuss further several of the arguments by Luyssaert et al. [1], showing why a large additional mitigation contribution by European forests towards climate objectives is unrealistic, and offering a different view on the relative importance of biophysical vs. biogeochemical climate effects of forest management in the EU.

## Main text

Considering that the current carbon sink in the EU LULUCF sector is about 300 Mt CO_2_/year (about 400 MtCO_2_e/year for forests alone)—offsetting about 7% of total EU GHG emissions, with rather stable values in the last 25 years [5], reaching a 25% contribution would require (i) that the current LULUCF sink nearly doubles by 2030, something not supported by any peer-reviewed publication, and (ii) that this sink is entirely counted as a mitigation effort towards the EU 2030 target. This second point has never been seriously considered in the political debate, because it has long been recognized that the existing forest sink is not entirely a result of direct and recent mitigation actions, but instead largely due to historic management activities and the effects of environmental change [6, 7] (see Additional file 1: Section S2.1). Accordingly, in the recent EU LULUCF legislation [8] not all of the forest management sink will count toward the mitigation target. Instead, only the portion that will exceed a predefined science-based “forest reference level” benchmark will count [7, 9], reflecting the atmospheric impact of additional actions. In the event that the forest sink is smaller than this benchmark, then the corresponding accounted “debit” from forest management will need to be compensated for, through extra emission reductions in other land uses or in other GHG sectors, such as energy.

Furthermore, the EU climate legislation [10] has capped both the amount of possible “credits” from managed forest land (equal to 3.5% of 1990 emissions) and the maximum contribution from the LULUCF sector toward the EU target at 280 MtCO_2_e for the period 2021–2030. If this latter value is annualized (i.e., 28 MtCO_2_e/year), it corresponds to about 1% of the EU 2030 emission reduction target. Therefore, contrary to the assumption of Luyssaert et al., almost all of the EU mitigation effort in 2030 is expected to come from emission reductions from non-LULUCF sectors and only a very small part directly from LULUCF.

Forests may contribute to mitigation also indirectly, especially through the utilization of wood as an energy source in place of fossil fuels. When the harvesting of forest biomass for energy purposes is increased, a decrease in carbon stock is reported in the LULUCF sector whilst GHG emission savings appear in the energy sector. For the EU, these savings are currently estimated to be about 130 MtCO_2_e/year [11], relative to about 90 MtCO_2_e/year in 1990 (see Additional file 1: Section S2.2). Several studies suggest a larger future potential associated with additional sustainable harvest of EU wood for bioenergy [12]. However, since this additional harvest would temporarily lower the forest sink, the relevant question is which portion of this bioenergy potential can be realized without generating accounted debits in the forestry and consequently in the LULUCF sector by 2030. Based on various sources [7, 11, 13], and assuming no LULUCF debits, we estimate that EU forest-based bioenergy derived from additional harvest could save about 150 MtCO_2_e/year in 2030. Relative to the EU’s climate target under the Paris Agreement (reduction of about 2250 Mt CO_2_e/year from 1990 to 2030), the indirect contribution of EU forest-based bioenergy to the EU 2030 emission reduction target would realistically add another 3% ((150 − 90)/2250).

We fully share with Luyssaert et al. [1] the view that forest management strategies aiming at climate change mitigation should not focus solely on GHG emissions, but should consider also any robust evidence on the potential impact of biophysical effects. However, we think that the uncertainty of the findings by Luyssaert et al. [1] has not been adequately emphasized. Although the local and seasonal climate biophysical effects can be retrieved with some confidence—e.g. afforestation warms winter surface temperature (decreased albedo) and cools summer surface temperature (increased evapotranspiration) [14]—the net annual impact of combined local and non-local effects in temperate zones, such as most of the EU, is highly uncertain. This is because in temperate regions radiative and non-radiative effects have similar magnitude but opposite impacts on the mean annual temperature. As a result, observation-based assessments and models don’t agree on the magnitude, and often not even on the sign, of the net annual biophysical climate effects of forestry in temperate zones [15, 16]. Despite the good advancements in Luyssaert et al.’s model (e.g., in representing differences between tree species and stand structures), the net annual biophysical climate impact of forest management in the EU remains more uncertain than the net CO_2_ impact. Rather than emphasizing these crucial caveats, Luyssaert et al. [1] used their results on the combined biophysical and biogeochemical effects to challenge their perception of EU policy towards forestry and climate change.

If the aim is to encourage countries to start considering biophysical effects in their policies, more emphasis should be put on seasonal and local impact of biophysical effects of forest cover change, including synergies and trade-offs with a carbon-oriented management, rather than on the net annual biophysical climate impact at EU level. These seasonal and local impacts are less uncertain and more relevant in the context of changes in diurnal temperature excursions [17] and heat extremes [18], and therefore for our perception of climate change. Concrete and accessible tools should be developed to allow countries to assess themselves the biophysical effects of different forest management scenarios [19]. Furthermore, model projections should be complemented with observational evidences, and a comprehensive communication of the uncertainty and range of applicability of the scientific findings is required to gain credibility in the policy domain.

Irrespective of the high uncertainty of biophysical effects on climate, the argument by Luyssaert et al. [1], that efforts for enhancing the CO_2_ sink from forest management are counterbalanced by negative biophysical climate effects—resulting in a “zero-sum” climate outcome, could be interpreted as forest management not being important to fight climate change. We think that would be a wrong conclusion. In fact, the recent inclusion of forests into the EU 2030 economy-wide climate targets [8] represents a key incentive for identifying the country-specific optimal mix, in terms of overall GHG balance, between strategies focused on conserving and/or enhancing the sink, as explicitly requested by the Paris Agreement (Art. 5), and strategies focused on using more wood to reduce emissions in other GHG sectors (including both energy and material substitution [20]). Without political “sticks and carrots” on GHG emissions, i.e. if forests were excluded from climate change mitigation strategies, there would be no incentive for conserving the current forest CO_2_ sink, and no disincentive for a possible over-use of forest resources (e.g., for bioenergy purposes), which could drastically reduce the current CO_2_ sink.

## Conclusions

In conclusion we argue that, while biophysical effects are clearly important on the local and seasonal climate, the net annual biophysical climate impact of forest management in Europe remains more uncertain than the net CO_2_ impact. Therefore, in our view, the conclusion of Luyssaert et al. [1] that the efforts for enhancing the CO_2_ sink from forest management at EU level are counterbalanced by negative biophysical climate effects is uncertain and premature. Furthermore, we show that the GHG mitigation contribution by forests towards EU 2030 climate objectives is expected to be small, but yet strategically important. Although the original mistake by Luyssaert et al. [1] on the expected large contribution of EU forests toward climate targets has been corrected, it reflects a misunderstanding of the policy context. These types of misunderstandings should be avoided, especially in high-visibility journals, because they create confusion in the debate on how forests may contribute to climate targets, such as the newly started discussion on the EU 2050 GHG strategy [21]. They also risk distracting the attention from the key intended message of the paper, hampering the prospect that biophysical effects of forest management—recently subject of a rising interest [22]—are seriously considered by policy makers. We hope that the clarifications provided here will foster a more correct understanding of the realistic role of forests within the EU climate targets and under the Paris Agreement [23], and encourage a more constructive dialogue between the scientific community and policy makers.

## Additional file

**Additional file 1.** The contribution of LULUCF to the countries' climate pledges made in Paris and, more specifically, the expected contribution of forests to meet EU 2030 the climate targets, including an analysis of forest-based bioenergy.

## Data Availability

The data supporting our conclusions on the contribution of forests to the EU climate targets are available either in the paper itself or in the papers listed in the references. Additional data may be requested from the corresponding author.

## Abbreviations

EU: European Union
GHG: greenhouse gases
LULUCF: land use, land-use change and forestry
