# Supplementary material for: On the realistic contribution of European forests to reach climate objectives
Source: Carbon Balance Manag. 2019 Jun 14;14:8. doi: 10.1186/s13021-019-0123-y (PMC7227057; doi:10.1186/s13021-019-0123-y)
Supplement: Supplementary file 1 — Additional file 1. The contribution of LULUCF to the countries' climate pledges made in Paris and, more specifically, the expected contribution of forests to meet EU 2030 the climate targets, including an analysis of forest-based bioenergy. [file 13021_2019_123_MOESM1_ESM.docx]

**Additional information**

**TITLE: On the realistic contribution of European forests to reach climate objectives**

Giacomo Grassi^1^*, Alessandro Cescatti^2^, Robert Matthews^3^, Gregory Duveiller^4^, Andrea Camia^5^, Sandro Federici^6^, Jo House^7^, Nathalie de Noblet-Ducoudré^8^, Roberto Pilli^9^, Matteo Vizzarri^10^

1. Giacomo Grassi, European Commission, Joint Research Centre, 21027 Ispra (VA), Italy. [giacomo.grassi@ec.europa.eu](mailto:giacomo.grassi@ec.europa.eu)
2. Alessandro Cescatti, European Commission, Joint Research Centre, 21027 Ispra (VA), Italy. Alessandro.Cescatti@ec.europa.eu
3. Robert Matthews, Forest Research, Alice Holt Lodge, Farnham GU10 4LH, United Kingdom, Robert.Matthews@forestry.gov.uk
4. Gregory Duveiller, European Commission, Joint Research Centre, 21027 Ispra (VA), Italy. Gregory.Duveiller@ec.europa.eu
5. Andrea Camia, European Commission, Joint Research Centre, 21027 Ispra (VA), Italy. [Andrea.Camia@ec.europa.eu](mailto:Andrea.Camia@ec.europa.eu)
6. Sandro Federici, Climate and Environment Division, FAO, 00153 Rome, Italy [sandro.federici@gmail.com](mailto:sandro.federici@gmail.com) ‎
7. Jo House, Cabot Institute, Department of Geographical Sciences, University of Bristol, Bristol BS8 1SS, UK, [Jo.House@bristol.ac.uk](mailto:Jo.House@bristol.ac.uk)
8. Nathalie de Noblet-Ducoudré, Laboratoire des Sciences du Climat et de l'Environnement LSCE/IPSL, Unité mixte CEA-CNRS-UVSQ, Université Paris-Saclay, F-91191 Gif-sur-Yvette, France, [nathalie.de-noblet@lsce.ipsl.fr](mailto:nathalie.de-noblet@lsce.ipsl.fr)
9. Roberto Pilli, European Commission, Joint Research Centre, 21027 Ispra (VA), Italy. Roberto.Pilli@ec.europa.eu
10. Matteo Vizzarri, European Commission, Joint Research Centre, 21027 Ispra (VA), Italy. Matteo.Vizzarri@ec.europa.eu

* corresponding author

**S1. The global contribution of LULUCF to the climate pledges made in Paris**

A comprehensive analysis of the global contribution of the Land Use, Land-Use Change and Forestry (LULUCF) sector to the countries’ climate pledges made at the Paris Climate Change Conference in December 2015^1^ has been done by Grassi et al.^2^. Another analysis, with broadly similar results, has been conducted by Forsell et al.^3^.

The figure 1 in the main text, in combination with Additional table S1 below, shows that globally LULUCF is expected to provide up to a quarter of the greenhouse gas (GHG) emission reductions planned by countries. Realizing and tracking this mitigation potential require more transparency in countries’ pledges and enhanced science-policy cooperation to increase confidence in numbers^2,4^. This global-level contribution mainly comes from the expected reduction of emissions from deforestation and forest degradation in Brazil and Indonesia.

**Table S1.** Information on Nationally Determined Contributions of the countries shown in Figure 1, based on Grassi et al.^2^. For further details, see Fig. 4b, supplementary section 2 and supplementary Table 5 in Grassi et al.^2^.

|  | **Information from Nationally Determined Contributions** | | | | | |
| --- | --- | --- | --- | --- | --- | --- |
|  | **Reference total GHG emissions** | | **Target** | | | **Planned total GHG emissions reduction**  **Gt CO_2_/y** |
|  | **year** | **Gt CO_2_/y** | **year** | **Max reduction**  (relative to reference year) | |  |
| **Brazil** | 2005 | 2.1 | 2030 | -43% | | -0.9 |
| **Indonesia** | BAU 2030 | 2.9 | 2030 | -41% | | -1.2 |
| **Russia** | 1990 | 3.9 | 2030 | -30% | | -1.2 |
| **EU** | 1990 | 5.6 | 2030 | -40% | | -2.3 |
| **Other countries** |  | 55.4 |  | |  | -10.3 |
| **All countries** |  | 69.9 |  | |  | -15.8 |

**S2. The contribution of EU forests to meet the climate targets**

**S2.1. Assessing the greenhouse gas mitigation by forest management**

Assessing the GHG mitigation outcomes in the forest sector is more complex than in other GHG sectors (e.g., energy, agriculture). This is because it can be hard to disentangle the simultaneous natural and anthropogenic processes that determine forest-related fluxes^5^. Moreover, unlike in other sectors, future emissions and removals in forests can change over time as a result of forest characteristics such as age-class distributions, which are largely determined by past forest management and natural disturbances^6^.

Under the United Nations Framework Convention on Climate Change (UNFCCC), this complexity has been addressed through a distinction between “reporting” and “accounting” of GHG fluxes, which is unique for the LULUCF sector^7,8^.

“Reporting” refers to the inclusion of estimates of anthropogenic GHG fluxes in national GHG inventories, following the methodological guidance provided by the Intergovernmental Panel on Climate Change (IPCC). The GHG inventories reported under the UNFCCC should, in principle, aim to reflect “what the atmosphere sees” in “managed lands”^4,9^, within the limits imposed by the method used and the available data. These inventories include the carbon stock changes in all the carbon pools, including living biomass, dead organic matter, soil and harvested wood products.

In the context of mitigation targets (e.g. under the Kyoto Protocol, the Paris Agreement and the EU 2030 climate targets), “accounting” refers to the comparison of emissions and removals with respect to a target and quantifies progress toward this target. Targets are typically expressed relative to the emissions in a base year (or a baseline/benchmark), thus the accounted mitigation outcomes should reflect genuine deviations from the activities generating emissions in the base year (or baseline/benchmark). For the LULUCF sector, specific “accounting rules” may be applied to filter reported flux estimates with the aim to better quantify the results of mitigation actions. LULUCF accounting then produces “debits” or “credits” (i.e. extra emissions or extra emission reductions, respectively) that count toward the target.

In the context of the recent EU LULUCF legislation^10^, the forest management mitigation (i.e. the contribution of managed forests to the EU climate targets in the period 2021-2030) will be determined by comparing the actual forest sink to a predetermined science-based projected benchmark (called “forest reference level”), which reflects the continuation of the historical forest management practices taking into account the dynamic impacts of forest aging^5,11^. If the actual sink falls below this reference level, the corresponding accounting “debit” will need to be compensated for, through extra emission reduction efforts in other GHG sectors, such as transport, agriculture or energy. Therefore, irrespective of the potential evolution of the forest sink^12^, if the contribution of forests to the EU target has to be assessed, the specific forest accounting rules (such as forest reference level) have to be fully taken into account. Once this is done, the contribution from forests to the EU 2030 target appears relatively small^5,13^, as discussed in the main text.

Beyond the “direct” forest GHG mitigation described above, reflected in the LULUCF sector, forests may contribute also “indirectly”, through the utilization of wood as an energy source and the substitution of energy intensive materials with durable long life wood products. Here we consider this contribution as “indirect” because it generates GHG savings in other GHG sectors, such as energy and buildings, but not directly in the LULUCF sector.

While the GHG savings from material substitution are addressed elsewhere^14^, in the following section we illustrate the methodology used to estimate the likely contribution of forest-based bioenergy to the EU 2030 climate target, as reported in the main text.

**S2.2.** **Estimating the contribution of forest-based bioenergy to the EU 2030 climate target**

The results presented in support of this comment are not intended to provide a comprehensive assessment of the GHG savings associated with the use of wood for energy purposes in the European Union (EU, including 28 Member States). Rather, we aim to provide an order of magnitude of the expected relative additional contribution of EU wood bioenergy to the EU 2030 climate targets. In doing so, we took into account the new EU LULUCF legislation^10^, and the accounting rules for forest management, i.e. the forest reference level reflecting the continuation of the historical forest management practices^5,11^. If the future sink falls below this reference level, the corresponding accounting “debit” from LULUCF will need to be compensated for, through extra emission reduction efforts in other GHG sectors.

In other words, we have tried to address the question: approximately how much of the planned 40% EU GHG emission reduction in 2030 vs. 1990 can be expected to come from an increase in the use of EU domestic wood production utilised for energy purposes in the same period, without generating accounting debits in the forest (and thus LULUCF) sector? To this aim, below we describe a systems analysis that we made of the essential elements of wood industry resource supply and utilization chains, referring to wood production statistics for the EU.

The Eurostat/UNECE/FAO/ITTO Joint Forest Sector Questionnaire (JFSQ) forest products datasets^15^, available through the FAOSTAT database^16^, provide data on the production and international trade of many commodities, including forestry products. These data can be used to estimate the quantity of wood produced and used for energy purposes within the EU.

The database contains data reported for each year on the roundwood produced by countries, including those countries comprising the EU. The reported roundwood production is also disaggregated into four categories of primary products, i.e. (i) sawlogs and veneer logs, (ii) pulpwood, (iii) other industrial roundwood and (iv) wood fuel (including wood for charcoal). These categories are also reported separately for coniferous and non-coniferous wood.

Although it may be assumed that all the material in the category of wood fuel is used for energy purposes, this does not represent all of the wood used for this purpose. This is because, in addition to wood harvested specifically for energy purposes, wood in other categories includes a significant proportion that is utilised for energy. When other categories of roundwood are processed into sawnwood, paper, card, panelboards, fencing products, etc., residues are generated as by-products in the form of offcuts, bark, slabwood, chips, sawdust and other wood particles. Hence, in order to estimate the total use of roundwood for energy purposes, it is necessary to estimate the proportion of industrial residues generated from other product categories, and of these the proportion utilised for energy. Since 1992, the production of chips, particles and residues has been reported explicitly as a separate category in FAOSTAT data, however, these categories were not reported in 1990. Furthermore, a significant proportion of this material is burned for process heat or power within sawmills and other production plants (and therefore not traded), so it is likely that the quantities reported under this category represent significant underestimates. Hence, the estimation of the quantities of total wood production in the EU used for energy purposes required the analysis of the subsequent utilisation of the four categories of roundwood described above. This analysis involved a number of assumptions as stated below.

Firstly, FAOSTAT roundwood statistics are reported as under-bark volumes (in cubic metres). For the purposes of this assessment, the reported roundwood statistics were converted to equivalent over-bark volumes, by assuming that bark represents an additional 15% of the under-bark roundwood volume of both coniferous and non-coniferous roundwood produced in the EU. However the considerable variation between tree species and within individual trees should be noted.

It was further assumed that 50% of the over-bark sawlog volume is converted into sawnwood with the remainder becoming residues in the form of chips (10%) and other types of residue (40%). For the pulpwood category, it was assumed that 85% of the over-bark volume is utilised for wood-based panels, paper and card, fencing etc., with 5% of the volume becoming residues in the form of chips with a further 10% becoming other residues. For the category of other industrial roundwood, it was assumed that 75% of the over-bark volume is utilised for wood-based panels, paper and card, fencing etc., with 5% of the volume becoming chips and a further 20% becoming other residues.

Having estimated the generation of chips and residues from the (non-wood fuel) roundwood categories, it was then assumed that 50% of the chips and 75% of the other residues are used for energy purposes.

Additional Table S2 shows estimates derived according to the methods described above for the total wood volume produced in the EU and used for energy purposes, in the years 1990 and 2015.

**Table S2. Estimates of wood volume produced in the EU (including 28 countries) and used for energy purposes**

| Year | Wood volume (Mm^3^) | | |
| --- | --- | --- | --- |
|  | Wood fuel^1^ | Other categories^2^ | Total |
| 1990 | 74 | 90 | 164 |
| 2015 | 125 | 107 | 232 |

1. Wood production reported as “wood fuel” in FAO statistics for the EU28 plus bark (15%)
2. Wood production in other FAO categories used for energy purposes, estimated as described above (including bark).

An estimate of the GHG emissions mitigated by the utilisation of wood produced in the EU used for energy purposes in the years 1990 and 2015 was then derived.

First, for each year, the result for total wood volume in Supplementary Table 1 was converted to an estimate of wood mass in units of oven-dry tonnes (odt). A conversion factor of 0.435 odt per m^3^ was conversion, based on assumptions about the wood density of coniferous and non-coniferous wood and the contributions made by these types of wood to total production. This gave estimates of 73 and 101 Modt respectively for the years 1990 and 2015. These results were estimated by an assumed emissions displacement factor of 1.32 tCO_2_-equivalent odt^-1^ (or roughly 0.57 tCO_2_-equivalent m^-3^), giving results for GHG emissions mitigated in 1990 and 2015 of 94 and 133 MtCO_2_-equivalent, respectively. The assumed GHG emissions displacement factor was based on LCA data and results reported by Matthews et al.^17^, and involved assuming a counterfactual energy source of oil. This leads to estimates of displaced GHG emissions that are greater than if a counterfactual of natural gas were to be assumed, but less than would be the case for an assumed counterfactual of coal.

We then combined the numbers above for 2015 with the additional EU-level harvest expected by 2030 under the continuation of historical forest management practices, i.e. consistent with the “forest reference level” accounting as described in the EU legislation and assuming no LULUCF debits. This additional harvest was estimated from various sources^5,12,17^, and ranges between about 40 and 70 additional Mm^3^ in 2030 relative to 2015. By considering the same share on energy vs. non-energy wood as documented historically^10^ and the conversion factors described above, the expected potential GHG emissions savings associated with forest-based bioenergy are estimated around 153 MtCO_2_ in 2030.

Therefore, relative to the EU’s climate target under the Paris Agreement (reduction of about 2250 Mt CO_2_e/y, i.e. from about 5650 Mt CO_2_e/y in 1990 to about 3400 Mt CO_2_e/y in 2030), the indirect contribution of EU forest-based bioenergy to the EU 2030 emission reduction target would realistically add another 3% ((153-94)/2250).

Note that, for the purpose of this comment, we do not discuss the specific GHG savings from various bioenergy pathways, extensively discussed elsewhere^18^.

It should be acknowledged that large uncertainties exist on the forest-based bioenergy data for 1990, and that different approaches (including different methods to disaggregate available statistics) may lead to different estimates of the amount of wood used for energy purposes.

As example of a different approach, Camia et al.^19^ performed a comprehensive analysis of wood flows within the forest sector, covering non-energy as well as energy uses of harvested wood, trade in wood commodities and reconciliation of estimates with other available data sources on wood energy use within the EU. They estimated EU wood resource balance sheets and woody biomass flows using both forest-based sector statistics (from the FAOSTAT database described above) and energy-related statistics such as the UNECE/FAO Joint Wood Energy Enquiry (JWEE)^20^ and the EU Member States national renewable energy action plans (NREAPs) Progress Reports^21^. For the year 2015, their findings show that energy statistics imply a significantly greater amount of forest-based bioenergy (more than 400 Mm^3^) compared to forest-based sector statistics^19^. This discrepancy between different statistics, equivalent to more than 100 Mm^3^ at EU level in 2015, is the amount of wood likely unaccounted from the source side, and which would be needed to explain the material and energy produced reported from the use side (see Camia et al.^19^ for details). Using data from Camia et al.^19^, the contribution of EU forest-based bioenergy to the EU 2030 climate targets would slightly increase, reaching about 4% of the foreseen total EU GHG emission reduction.

Furthermore, it should be also noted that the recast of the EU Renewable Energy Directive^22^ introduces new risk-based requirements for forest biomass in order ‘to minimise the risk of using unsustainable forest biomass production’ and alignment with LULUCF requirements, with the aim of ensuring proper carbon accounting.

Despite the uncertainties illustrated above, we believe that the results provided here represent robust evidence to support the message that, in fact, the additional contribution of EU forest-based bioenergy to the EU 2030 climate targets should be expected to be relatively small, i.e. in the order of 3 to 4% of the foreseen total EU GHG emission reduction.

**References (Additional Information)**

1. UNFCCC (United Nations Framework Convention on Climate Change). INDCs as communicated by Parties. Available at: https://www4.unfccc.int/sites/submissions/indc/Submission Pages/submissions.aspx.

2. Grassi, G. *et al.* The key role of forests in meeting climate targets requires science for credible mitigation. *Nat. Clim. Chang.* **7**, 220–226 (2017).

3. Forsell, N. *et al.* Assessing the INDCs’ land use, land use change, and forest emission projections. *Carbon Balance Manag.* **11**, 26 (2016).

4. Grassi, G. *et al.* Reconciling global-model estimates and country reporting of anthropogenic forest CO2 sinks. *Nat. Clim. Chang.* **8**, 914–920 (2018).

5. Grassi, G., Pilli, R., House, J., Federici, S. & Kurz, W. A. Science-based approach for credible accounting of mitigation in managed forests. *Carbon Balance Manag.* **13**, 8 (2018).

6. Böttcher, H., Kurz, W. A. & Freibauer, A. Accounting of forest carbon sinks and sources under a future climate protocol—factoring out past disturbance and management effects on age–class structure. *Environ. Sci. Policy* **11**, 669–686 (2008).

7. UNFCCC (United Nations Framework Convention on Climate Change). Land Use, Land-Use Change and Forestry (LULUCF). Available at: https://unfccc.int/topics/land-use/workstreams/land-use--land-use-change-and-forestry-lulucf.

8. Iversen, P., Lee, D. & Rocha, M. *Understanding Land Use in the UNFCCC*. (2014). http://www.climateandlandusealliance .org/wp-conte nt/uploa ds/2015/08/Under stand ing_Land_Use_in_the_UNFCC C.pdf.

9. IPCC. *2006 IPCC Guidelines for National Greenhouse Gas Inventories*. (2006).

10. EU (European Union). *Regulation (EU) 2018/841 of the European Parliament and of the Council of 30 May 2018 on the inclusion of greenhouse gas emissions and removals from land use, land use change and forestry in the 2030 climate and energy framework, and amending Regulation (EU) No 525/2013 and Decision No 529/2013/EU (Text with EEA relevance)*. (2018).

11. Forsell, N. *et al.* *Guidance on developing and reporting Forest Reference Levels in accordance with Regulation (EU) 2018/841*. (2018).

12. Nabuurs, G.-J., Arets, E. J. M. M. & Schelhaas, M.-J. Understanding the implications of the EU-LULUCF regulation for the wood supply from EU forests to the EU. *Carbon Balance Manag.* **13**, 18 (2018).

13. EC (European Commission). *Commission Staff Working Document. Impact assessment accompanying the document Proposal for a Regulation of the European Parliament and of the Council on the inclusion of greenhouse gas emissions and removals from land use, land use change and forestry into the 2030 climate and energy framework and amending Regulation No 525/2013 of the European Parliament and the Council on a mechanism for monitoring and reporting greenhouse gas emissions and other information relevant to climate change*. (2016).

14. Rüter, S. *et al.* *ClimWood2030, Climate benefits of material substitution by forest biomass and harvested wood products: Perspective 2030 - Final Report*. (2016).

15. UNECE & FAO. Joint Forest Sector Questionnaire. Available at: https://www.unece.org/forests/forestsfpmonlinedata/jfsq.html.

16. FAO. FAOSTAT: Forestry Production and Trade. Available at: www.fao.org/faostat/en/#data/FO.

17. Matthews, R. *et al.* *Carbon impacts of biomass consumed in the EU: quantitative assessment. Final report, project: DG ENER/C1/427 Part A: Main Report*. (2015).

18. Agostini, A., Giuntoli, J. & Boulamanti, A. *Carbon accounting of forest bioenergy. Conclusions and recommendations from a critical literature review*. (2014).

19. Camia, A. *et al.* *Biomass production, supply, uses and flows in the European Union. First results from an integrated assessment*. (2018).

20. UNECE & FAO. Joint Wood Energy Enquiry. Available at: www.unece.org/forests/jwee.html.

21. EC (European Commission). EU Science Hub: National renewable energy action plans and progress reports data portal. Available at: https://ec.europa.eu/jrc/en/scientific-tool/nreap-data-portal.

22. EP (European Parliament). Legislative train schedule. Resilient Energy Union with a climate change policy. JD - Review of the Renewable Energy Directive 2009/28/EC to adapt it to the EU 2030 climate and energy targets. Available at: www.europarl.europa.eu/legislative-train/theme-resilient-energy-union-with-a-climate-change-policy/file-jd-renewable-energy-directive-for-2030-with-sustainable-biomass-and-biofuels.
